# Supplementary material for: MetaQTL: a package of new computational methods for the meta-analysis of QTL mapping experiments
Source: BMC Bioinformatics. 2007 Feb 8;8:49. doi: 10.1186/1471-2105-8-49 (PMC1808479; doi:10.1186/1471-2105-8-49)
Supplement: Additional File 7 — MetaQTL Package : jar file and tutorial. This Zip archive contains both the MetaQTL JAR file and the files of the tutorial. [file 1471-2105-8-49-S7.zip › org.inra.metaqtl/doc/org/metaqtl/graph/GraphShape.html]

GraphShape


|  |  |  |
| --- | --- | --- |
| **Overview** \| **Package** \| **Class** \| **Use** \| **Tree** \| **Deprecated** \| **Index** \| **Help** | |  |
| **PREV CLASS**   **NEXT CLASS** | **FRAMES**    **NO FRAMES**     **All Classes** |  |
| SUMMARY: NESTED \| FIELD \| CONSTR \| METHOD | DETAIL: FIELD \| CONSTR \| METHOD |  |


---


## org.metaqtl.graph Class GraphShape

```
java.lang.Object
  org.metaqtl.graph.GraphShape
```

**Direct Known Subclasses:**: ChromShape, QtlShape

---

``` public abstract class GraphShape extends java.lang.Object ```

---

| **Field Summary** | |
| --- | --- |
| `protected  double` | `height` |
| `protected  double` | `width` |
| `protected  double` | `x` |
| `protected  double` | `y` |


**Constructor Summary**

`GraphShape()`


| **Method Summary** | |
| --- | --- |
| `abstract  void` | `buildShape(java.awt.Graphics2D graph, java.lang.Object object)` |
| `abstract  void` | `draw(java.awt.Graphics2D graph)`             Draws the shape. |
| `double` | `getHeight()` |
| `double` | `getWidth()` |
| `abstract  double` | `getYMax()` |
| `abstract  double` | `getYMin()` |

**Methods inherited from class java.lang.Object**

`clone, equals, finalize, getClass, hashCode, notify, notifyAll, toString, wait, wait, wait`

**Field Detail**

### x

```
protected double x
```

---


### y

```
protected double y
```

---


### height

```
protected double height
```

---


### width

```
protected double width
```


**Constructor Detail**

### GraphShape

```
public GraphShape()
```


**Method Detail**

### buildShape

```
public abstract void buildShape(java.awt.Graphics2D graph,
                                java.lang.Object object)
```

---


### draw

```
public abstract void draw(java.awt.Graphics2D graph)
```

:   Draws the shape.

    :   **Parameters:**: `graph` -

---


### getHeight

```
public double getHeight()
```

---


### getWidth

```
public double getWidth()
```

---


### getYMin

```
public abstract double getYMin()
```

---


### getYMax

```
public abstract double getYMax()
```


---


|  |  |  |
| --- | --- | --- |
| **Overview** \| **Package** \| **Class** \| **Use** \| **Tree** \| **Deprecated** \| **Index** \| **Help** | |  |
| **PREV CLASS**   **NEXT CLASS** | **FRAMES**    **NO FRAMES**     **All Classes** |  |
| SUMMARY: NESTED \| FIELD \| CONSTR \| METHOD | DETAIL: FIELD \| CONSTR \| METHOD |  |


---
